# No-touch technique for saphenous vein graft harvesting in coronary artery bypass surgery safely improves graft patency: a meta-analysis of randomized controlled trials

**DOI:** 10.1007/s12055-024-01788-5

**Published:** 2024-08-16

**Authors:** Tri Wisesa Soetisna, Ahmad Muslim Hidayat Thamrin, Mahardika Budjana Sutan Ilham, Marko Darmawan, Faris Maulana Irfan, Vicky Supit, Sugisman, Dudy Arman Hanafy, Amin Tjubandi, Dicky Aligheri Wartono, Wirya Ayu Graha

**Affiliations:** 1 Adult Cardiac Surgery Division, Department of Thoracic and Cardiovascular Surgery, Harapan Kita National Cardiovascular Center Hospital, S. Parman Street Cavling 87, Jakarta, 11420 Indonesia; 2 Department of Thoracic and Cardiovascular Surgery, Faculty of Medicine, University of Indonesia, Jakarta, 10430 Indonesia; 3 Faculty of Medicine, Syarif Hidayatullah State Islamic University, Haji Hospital, Jakarta, 13560 Indonesia

**Keywords:** No-touch, Vein graft harvesting, Outcome, Safety, Coronary artery bypass, Ischemic heart disease

## Abstract

**Objective:**

The no-touch (NT) technique for saphenous vein graft (SVG) harvesting has been gaining popularity as several trials have shown its superiority in maintaining graft patency. However, this technique’s clinical outcome and safety are still disputed and the results vary widely. The aim of this meta-analysis of randomized controlled trials (RCTs) was to assess the effectiveness and safety of this method.

**Methods:**

The Preferred Reporting Items for Systematic Reviews and Meta-Analyses (PRISMA) guidelines were conducted for this systematic review and meta-analysis. A comprehensive search of the literature was carried out with Embase, Scopus, and PubMed databases. The articles underwent extensive evaluation and analysis.

**Results:**

Six RCTs comparing the NT and conventional (CON) techniques were included. Primary outcomes were measured using graft occlusion. Graft failure rates and clinical outcomes including major adverse cardiac and cerebrovascular events (MACCE), all-cause death, myocardial infarction, repeat revascularization, and leg wound complications were evaluated as secondary outcomes. The NT technique significantly decreased graft occlusion (risk ratio (RR) = 0.58; 95% confidence interval (CI) = 0.46 to 0.72; *p* < 0.001) and failure (RR = 0.65; 95% CI = 0.54 to 0.77; *p* < 0.001). Safety analysis also showed no significant risk difference for clinical outcomes, and although significantly higher, leg complications in the NT technique are minor and avoidable.

**Conclusion:**

The NT technique increases long-term graft patency with no significant risk difference for clinical outcomes compared to the CON technique. However, the leg wound complications are significantly higher in the NT technique compared to the CON technique.

**Graphical Abstract:**

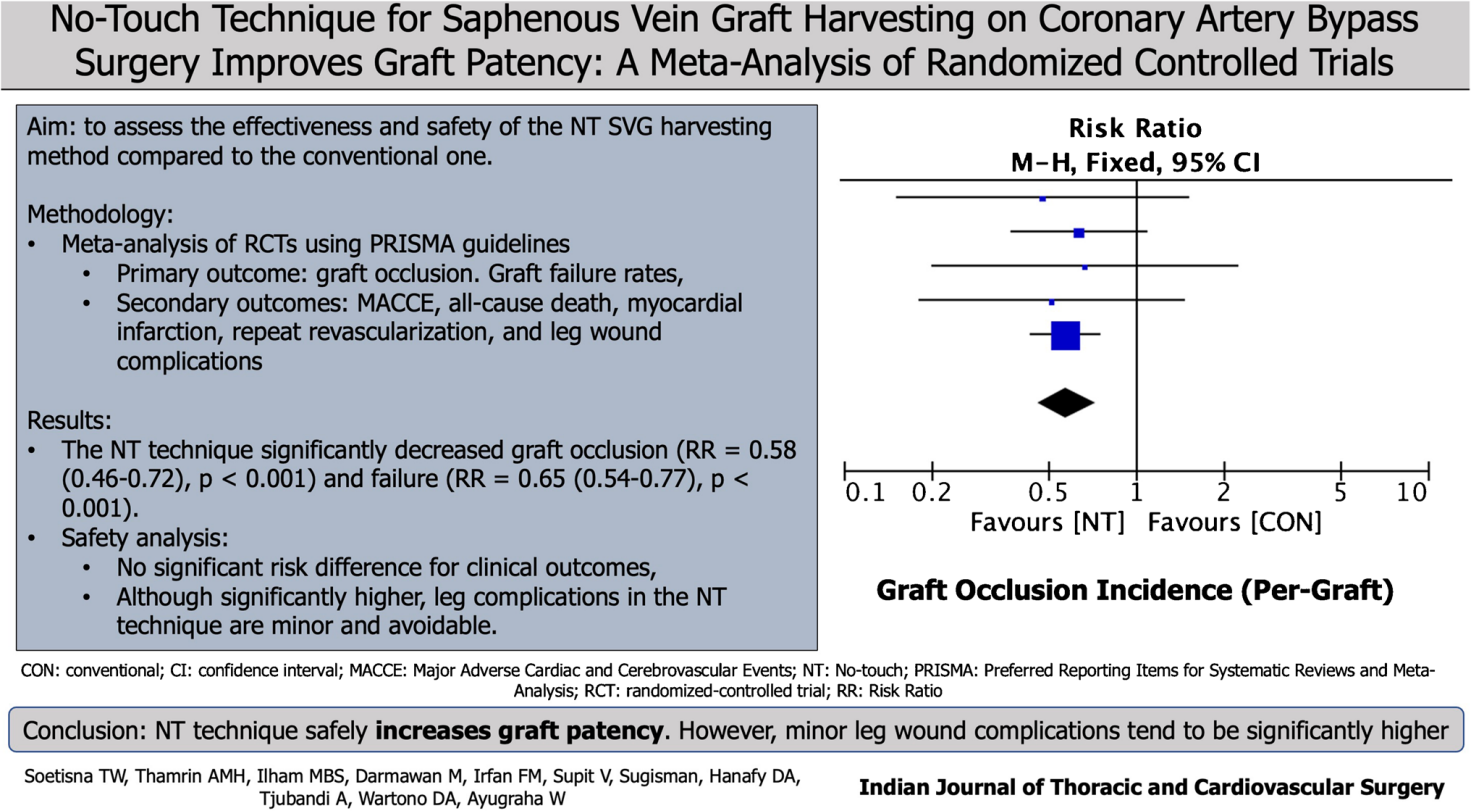

## Introduction

With over one million procedures performed annually, saphenous vein grafts (SVGs) continue to be the most commonly used graft for coronary artery bypass grafting (CABG) due to their ease of harvest [1, 2]. Unfortunately, these grafts exhibit higher rates of occlusion and graft failure compared to arterial grafts [3]. The endothelial damage associated with them has been widely recognized as one of the major hazards contributing to failure [4].

The no-touch (NT) technique is performed by leaving a layer of tissue around the vein to act as a cushion, resulting in an “untouched” SVG [5, 6]. This technique preserves the natural architecture of the perivascular fat pad, thereby reducing spasm, reducing high-pressure intraluminal distension, and leaving the endothelium intact [7]. The use of this technique for SVG harvesting can be traced back 30 years [5]. The result of the first randomized trial was reported in 2002 and showed that preserving the tissue around the saphenous vein was important for graft performance and patency [8]. Although some variables were not statistically significant, similar results were found in several trials, including a subsequent randomized controlled trial (RCT) [9–13]. The major adverse cardiac and cerebrovascular events (MACCE) study also showed no difference between the NT and conventional (CON) techniques [10, 12, 13]. However, several studies showed that leg wound complications were greater in the NT group than in the CON group [8, 10, 12, 13].

We performed a meta-analysis to pool the results of NT and CON SVG harvesting techniques. We selected the meta-analysis of RCT studies only to increase the scientific strength of the studies by knowing that several related RCTs with a large number of subjects have reported their results in the past 5 years. We compared graft patency, as well as clinical outcomes and leg infections to comprehensively evaluate whether the NT technique outweighs its risks.

## Materials and methods

### Eligibility criteria

The included studies were randomized trials conducted in patients undergoing CABG, comparing the outcomes of NT and CON vein harvesting techniques. Reviews, unpublished works, editor’s letters, abstracts, and studies in languages other than English were excluded from this analysis. Our study was registered in the a priori systematic review protocol registry in the International Prospective Register of Systematic Reviews (PROSPERO) with registration number CRD42023407651.

### Type of outcome measurement

Graft occlusion rate was the primary/main outcome measure. We also assessed graft failure rate and clinical outcomes: MACCE, death from any cause, myocardial infarction, and repeat revascularization along with complications from leg wounds. Graft occlusion was defined as the presence of string signs on angiography, while graft failure was defined as graft stenosis of more than 50% or occlusion.

### Search techniques and identification of studies

#### Information sources

This systematic review and meta-analysis used the Preferred Reporting Items for Systematic Reviews and Meta-Analyses (PRISMA) guidelines [14]. Throughout March and April of 2023, Embase, Scopus, and PubMed electronic databases were explored to search for literature. We restricted the language of the articles we found by applying language restrictions. We did not apply any publication time restrictions to our review.

#### Search protocol

The Patient/Population, Intervention, Comparison, Outcomes (PICO) model was used to formulate the study question. A pre-established PICO design was utilized to choose the pertinent studies that would be included in the review. The trial registrations and databases were all searched using the following keywords: (“no touch” OR “pedicled” OR “pedicle”) AND (“coronary artery bypass” OR “CABG”) AND (“trial” OR “randomized” OR “RCT”).

#### Data collection and analysis

Titles and abstracts were used to filter every search record. The studies were independently assessed by four authors (TWS, AMHT, MBSI, and MD) using both inclusion and exclusion criteria. First, those with irrelevant abstracts and titles were eliminated. Publications in languages other than English were immediately excluded. All authors then evaluated only full-text RCT studies that met the qualifying requirements. The details of the rationale for exclusion were recorded and communicated.

#### Data extraction and management

Data were extracted separately by three authors (TWS, AMHT, and MBSI) from a tabulation that included details on patient characteristics, treatments, study quality, and treatment outcomes [15]. A table was created for qualitative analysis that included information on authors, year of study publication, number of patients involved, pre-specified graft patency measures (graft occlusion rate and graft failure rate), clinical outcomes (MACCE, death from any cause, myocardial infarction, and repeat revascularization), and complications associated with foot wounds. For each outcome variable, we determined the risk ratio (RR) by extracting the number of events in each group. All data were entered into Review Manager software (RevMan), version 5.4, by four review authors (AMHT, TWS, MBSI, and MD) [16].

### Assessment of study quality and risk of bias in included studies

Using the standards outlined in the Cochrane handbook for systematic reviews of interventions for randomized studies—also known as Risk of Bias 2 (RoB 2) for randomized studies—the AMHT and TWS evaluated the risk of bias in each study that was part of the systematic review [15]. The authors then discussed the results of each reviewer’s assessment. Taking into account the overall risk of bias assessment, the results of the systematic review were interpreted using a risk of bias table and a summary of the biased aspects of the included studies [17, 18].

## Results

**Fig. 1:**
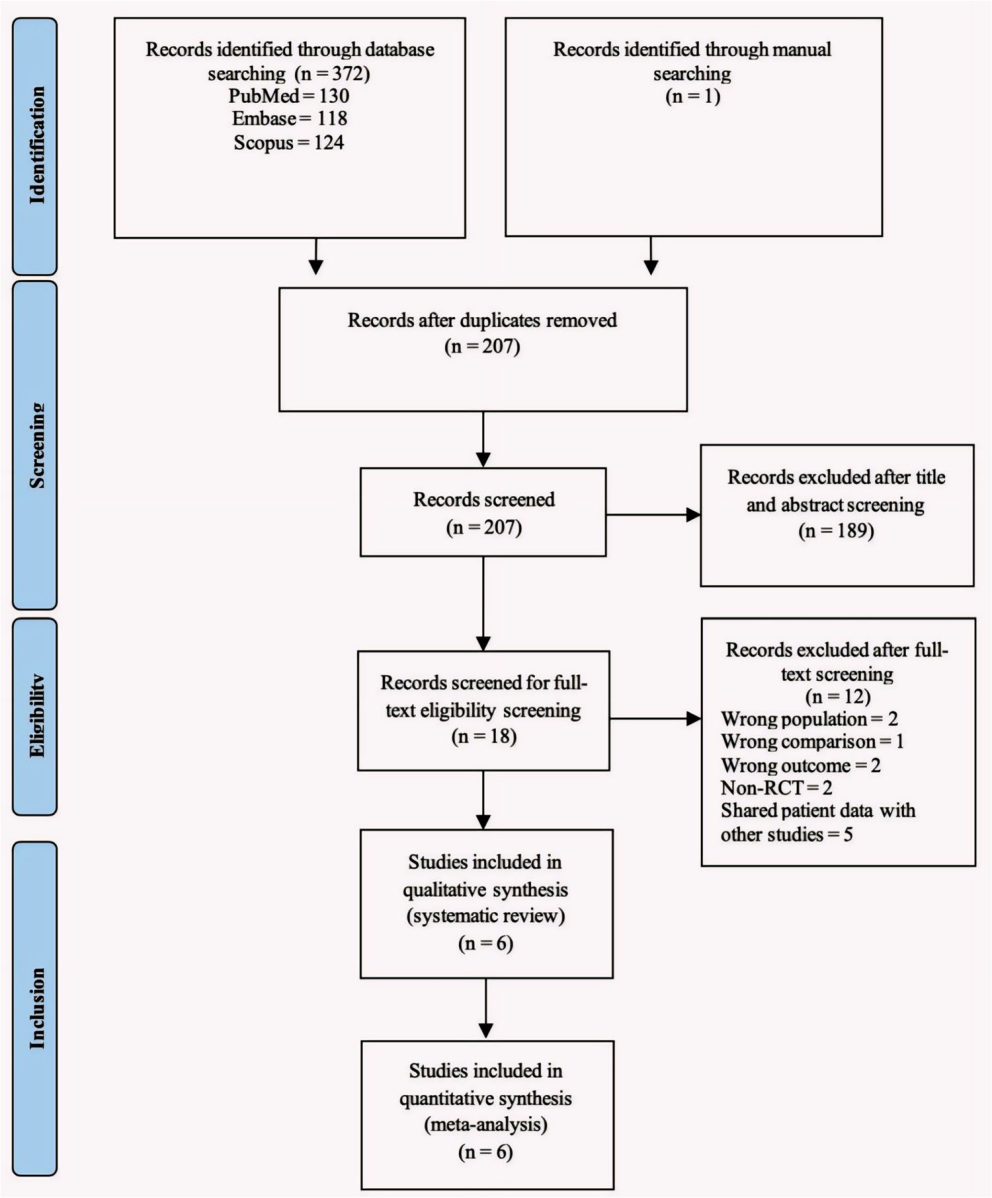
Preferred Reporting Items for Systematic Reviews and Meta-Analyses (PRISMA) guidelines flowchart

A total of 372 studies were selected and put through screening. After 18 of the RCTs were evaluated for eligibility, six of them were found to be suitable for the meta-analysis and were included in the systematic review. Figure 1 shows a graph of the assortment procedure.

### Demographic, preoperative, and intraoperative characteristics of the studies

A total of 3275 patients were included, with 1647 patients in the NT group and 1628 patients in the CON group in 6 RCTs. The majority of the study reports indicated that male participants comprised more than 70% of the subjects in their papers. The mean age varied across the reported studies. All studies reported longer operative time, cardiopulmonary bypass time, and aortic clamping time in the NT group. All studies used SVGs obtained from leg segments using an open technique [8, 10, 12, 13, 19, 20].

The follow-up period was time-driven and stretched from 3 months [20] to 18 months [8] with a median of 12 months [10, 12, 13, 19]. Three studies [8, 10, 19] used conventional coronary angiography as the graft assessment method, whereas three other studies [12, 13, 20] used computed tomography angiography (CTA) for evaluation. Table 1 displays a summary of the included papers.

**Table 1:** Summary of the studies included in the review

Author, year	Country	Number of patients		Number of vein grafts		Age (years, mean ± SD)		Surgical time (min, mean ± SD)		CPB time (min, mean ± SD)		Aortic cross clamp time (min, mean ± SD)		Follow-up period (months)	Outcome measurement method
		NT (male, *n*)	CON (male, *n*)	NT	CON	NT	CON	NT	CON	NT	CON	NT	CON		
Souza et al. 2002 [8]	Sweden	45 (48)	46 (40)	124	127	58 (6)	58 (6.8)	-	-	139 (26)	121 (32)	72 (18)	64 (15)	18	Coronary angiography
Pettersen et al. 2017 [10]	Norway	49 (45)	51 (33)	88	91	65 (6.9)	63.4 (7.1)	161 (27)	144 (24)	76 (21)	65 (18)	47 (14)	43 (15)	6	Coronary angiography
Deb et al. 2019 [12]	Canada (multi-center)	127 (106)	123 (113)	191	243	65.5 (9)	64.0 (8.2)	312 (96)	288 (78)	105 (37)	101 (37)	79 (35)	76 (35)	12	CTA
Tian et al. 2021 [13]	China (multi-center)	1325 (1042)	1313 (1027)	2317	2273	60.9 (8.4)	60.8 (8.0)	220 (16)	217 (16)	102 (36)	97 (29)	66 (26)	65 (24)	12	CTA
Angelini et al. 2021 [19]	UK	51 (46)	45 (41)	48	44	67.3	64.5	182 (160)	180 (165)	84 (63)	78 (67.5)	43.5 (32.5)	44 (37.5)	12	Coronary angiography
Hou et al. 2021 [20]	China	50 (46)	50 (47)	88	84	61 (8.7)	59.8 (7.8)	220 (188)	190 (175)	-	-	-	-	3	CTA

*CON*, conventional; *CPB*, cardiopulmonary bypass; *CTA*, computed tomography angiography; *min*, minutes; *NT*, no-touch; *SD*, standard deviation

### Primary outcome

#### Graft occlusion

**Fig. 2:**
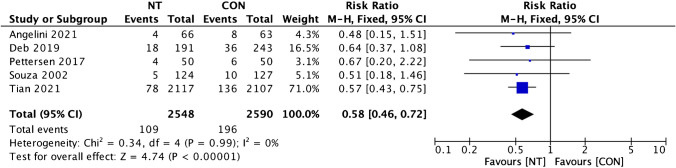
Forest plot of graft occlusion incidence (per graft) difference between NT and CON groups [8, 10, 12, 13, 19]. CI, confidence interval; CON, conventional; NT, no-touch

**Fig. 3:**
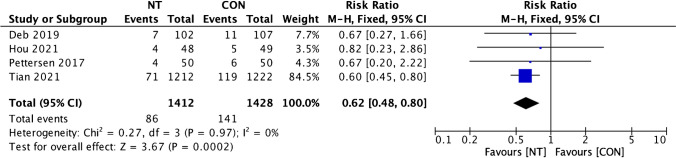
Forest plot of graft occlusion incidence (per patient) difference between NT and CON groups [10, 12, 13, 20]. CI, confidence interval; CON, conventional; NT, no-touch

We conducted both per-graft and per-patient analyses for graft evaluation. In total, 5138 vein grafts were evaluated: 2548 grafts from the NT group and 2590 grafts from the CON group. For the analysis of graft occlusion rate per graft, the heterogeneity rate was short (*I*^2^ = 0%), then the fixed effect model was used. Our examination of graft occlusion incidence per graft between the NT and CON groups revealed a remarkably lower incidence of graft occlusion in the NT group (RR = 0.58; 95% confidence interval (CI) = 0.46 to 0.72; *p* < 0.001) (Fig. 2). The same state was found in the investigation of per-patient graft occlusion rate which also revealed low heterogeneity (*I*^2^ = 0%) then fixed effect model was also used. Our evaluation of the disparity in the incidence of per-patient graft occlusion between the NT and CON groups also showed a notably lower incidence of graft occlusion in the NT group (RR = 0.62; 95% CI = 0.48 to 0.80; *p* < 0.001) (Fig. 3).

#### Clinical outcomes

**Fig. 4:**
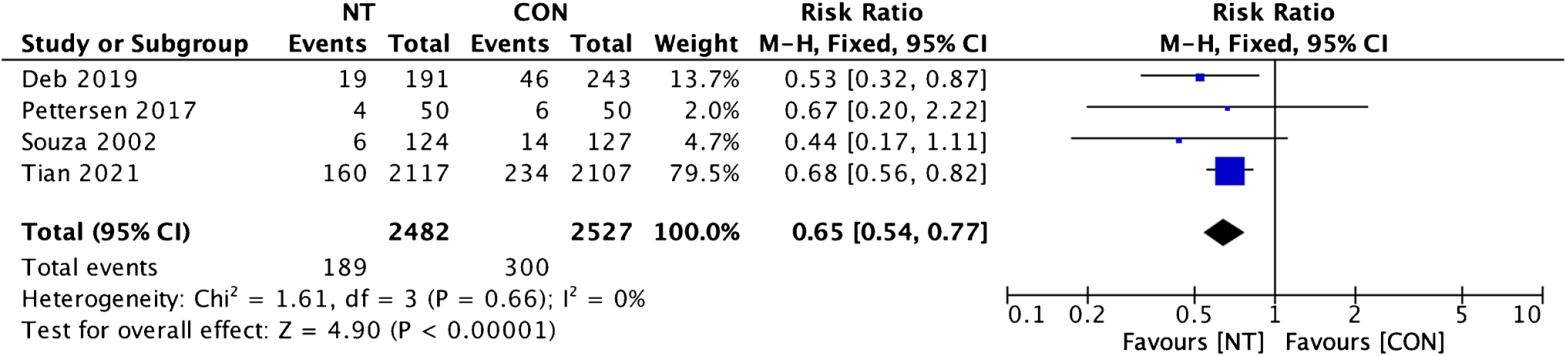
Forest plot of graft failure incidence (per graft) difference between NT and CON groups [8, 10, 12, 13]. CI, confidence interval; CON, conventional; NT, no-touch

**Fig. 5:**
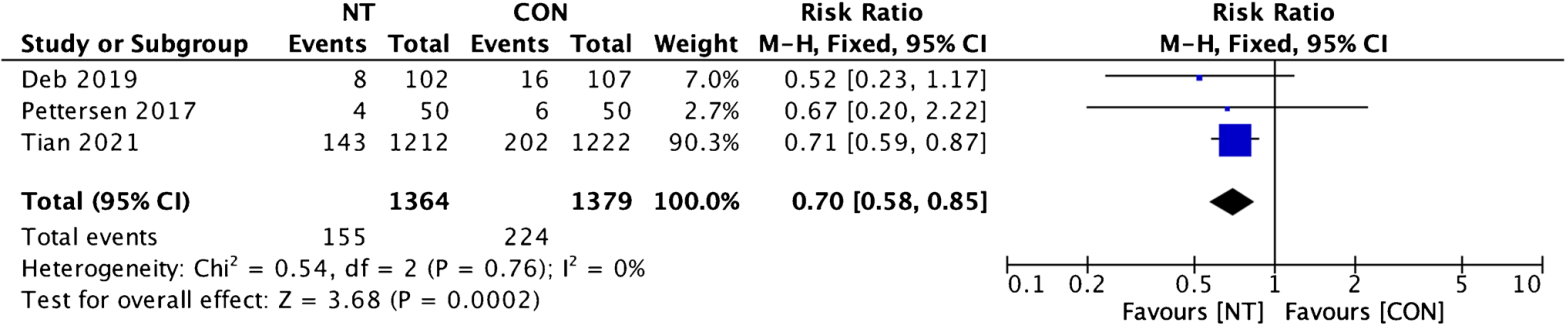
Forest plot of graft failure incidence (per patient) difference between NT and CON groups [10, 12, 13]. CI, confidence interval; CON, conventional; NT, no-touch

Graft failure rate per graft analysis showed low heterogeneity (*I*^2^ = 0%), and then a fixed effect model was used. Graft failure events per-graft disparity between groups showed a significantly lower event in the NT group (RR = 0.65; 95% CI = 0.54 to 0.77; *p* < 0.001) (Fig. 4). Per-patient graft failure rate investigation also showed low heterogeneity (*I*^2^ = 0%), then a fixed effect model was also used. Our analysis of per-patient graft failure events variance between the NT and CON groups also showed a remarkably lower occurrence of graft failure in the NT group (RR = 0.70; 95% CI = 0.58 to 0.85; *p* < 0.001) (Fig. 5).

**Fig. 6:**
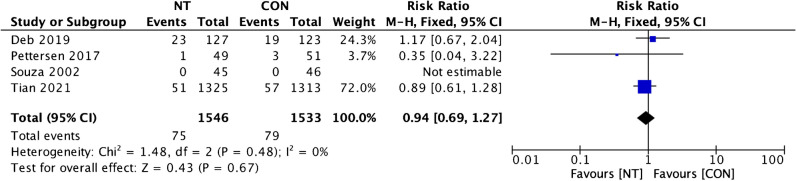
Forest plot of MACCE incidence difference between NT and CON groups [8, 10, 12, 13]. CI, confidence interval; CON, conventional; MACCE, major adverse cardiac and cerebrovascular events; NT, no-touch

**Fig. 7:**
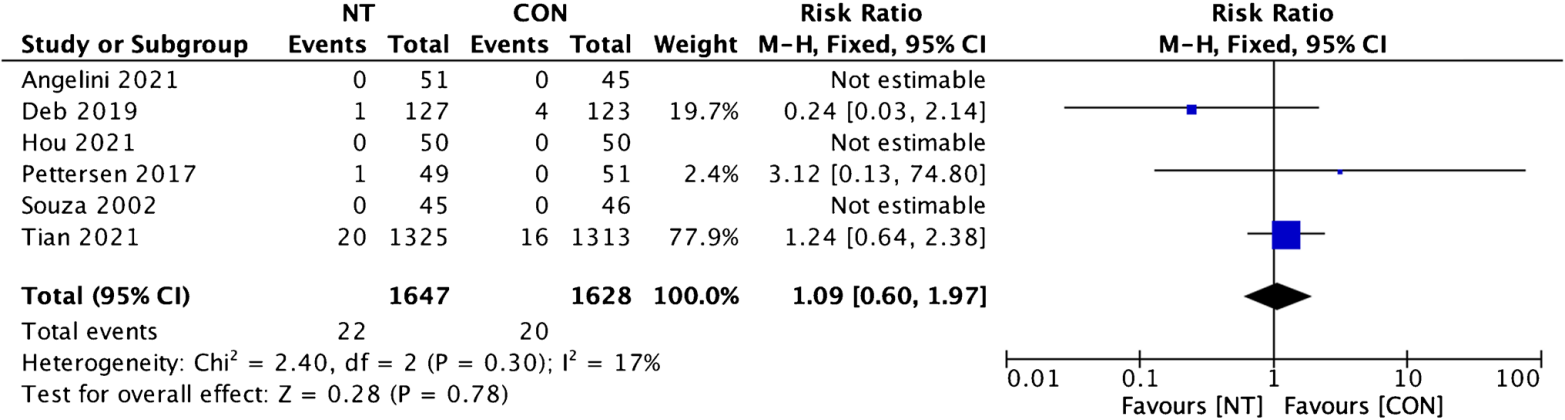
Forest plot of all-cause death incidence difference between NT and CON groups [8, 10, 12, 13, 19, 20]. CI, confidence interval; CON, conventional; NT, no-touch

**Fig. 8:**
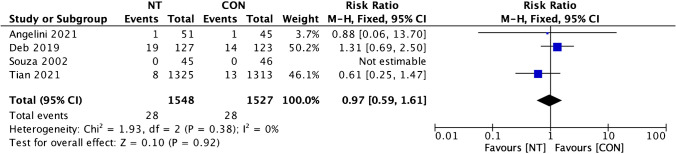
Forest plot of non-fatal myocardial infarction incidence difference between NT and CON groups [8, 12, 13, 19]. CI, confidence interval; CON, conventional; NT, no-touch

**Fig. 9:**
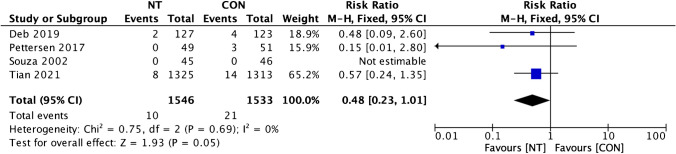
Forest plot of repeat revascularization incidence difference between NT and CON groups [8, 10, 12, 13]. CI, confidence interval; CON, conventional; NT, no-touch

Clinical outcomes comparison of the NT technique and the CON technique showed insignificant dissimilarity between the two groups. A favorable result for the NT group was found on MACCE incidence analysis from four trials, but it was statistically insignificant (RR = 0.94; 95% CI = 0.69 to 1.27; *p* = 0.67) (Fig. 6). Four studies’ evaluations of the incidence of all causes of death revealed a beneficial outcome for the CON group, but it was insignificant (RR = 1.09; 95% CI = 0.60 to 1.97; *p* = 0.78; Fig. 7). A favorable outcome for the NT group was also observed in the analysis of non-fatal myocardial infarction episodes from three investigations. However, the results were statistically insignificant (RR = 0.97; 95% CI = 0.69 to 1.61; *p* = 0.92) (Fig. 8). Another favorable outcome for the NT group was revealed by repeat revascularization events analysis from four trials, but it was also statistically insignificant (RR = 0.48; 95% CI = 0.23 to 1.01; *p* = 0.05) (Fig. 9).

### Secondary outcome (leg infection)

**Fig. 10:**
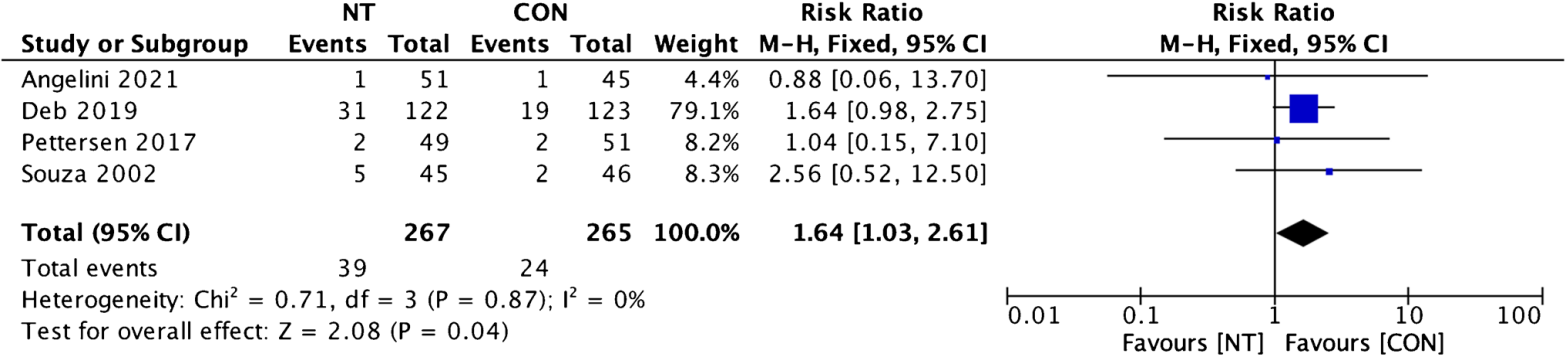
Forest plot of leg infection incidence difference between NT and CON groups [8, 10, 12, 19]. CI, confidence interval; CON, conventional; NT, no-touch

The evaluation outcomes for leg infections in both groups were reported in four studies [8, 10, 12, 19]. The results of the heterogeneity test indicated low heterogeneity (*I*^2^ = 0%), allowing us to choose a fixed-effect model. The examination of leg infection incidence produced favorable outcomes for the CON group and was statistically significant (RR = 1.64; 95% CI = 1.03 to 2.61; *p* = 0.04) (Fig. 10).

### Risk of bias analysis

**Fig. 11:**
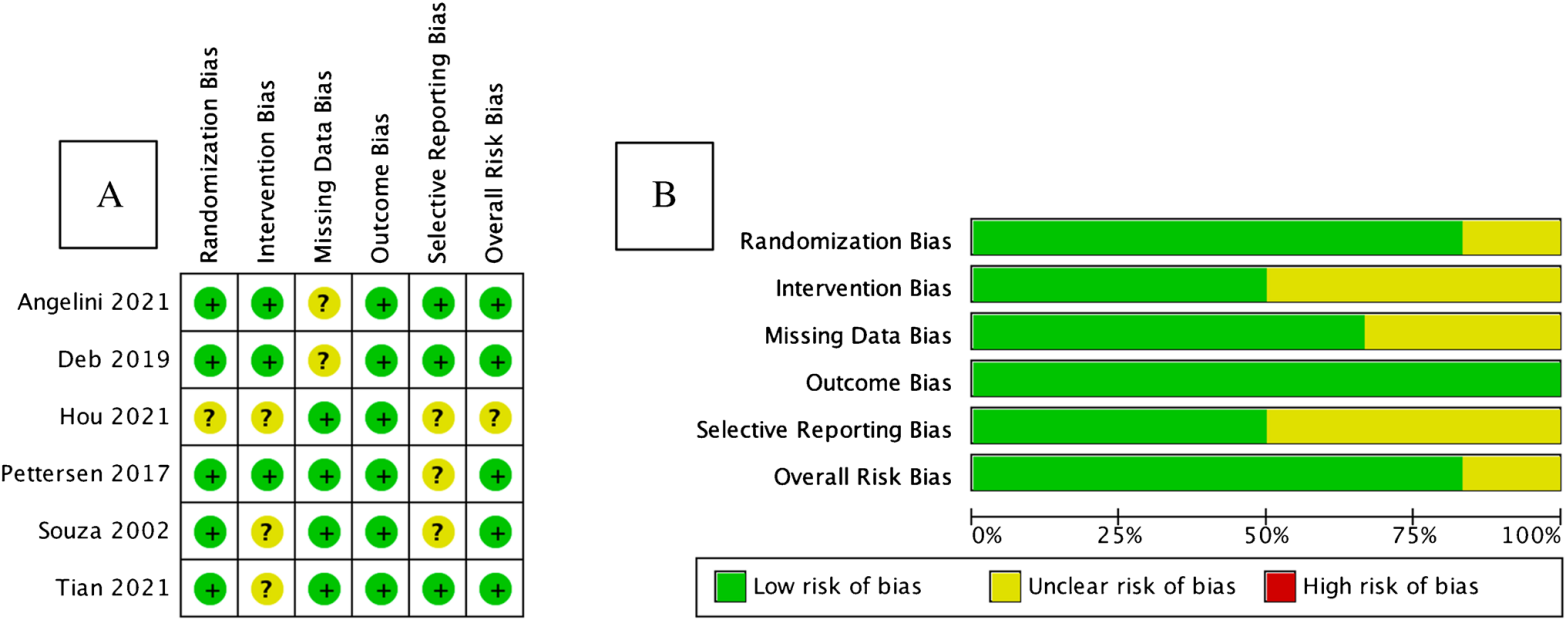
Results of RoB 2 assessment. **A** Risk of bias summary: review authors’ judgments about each risk of bias item for each included study using RoB 2 for randomized studies. **B** Risk of bias graph: review authors’ judgments about each risk of bias item presented as percentages across all included studies using RoB 2 for randomized studies [8, 10, 12, 13, 19, 20]. RoB 2, Risk of Bias 2

RoB 2 for randomized trials was utilized to assess the risk of bias in the included studies. The majority of the studies generally had a very low risk of bias. The result is displayed in Fig. 11.

## Discussion

Despite longer surgical times associated with NT, the study found significant reductions in graft occlusion and failure rates compared to conventional methods. The results were consistent across all the studies [8, 10, 12, 13, 19, 20], regardless of whether conventional coronary angiography [8, 10, 19] or CTA [12, 13, 20] was used to detect stenosis. Elmaghraby et al. demonstrated that, with an average limit of agreement of 0.8 (kappa value), the two modalities demonstrated good agreement in identifying significant vessel stenosis and total occlusion [21].

Clinical outcome evaluations showed no significant differences in MACCE, all-cause death, non-fatal myocardial infarction, and revascularization occurrences between the NT and CON techniques. However, from the study of Tian et al. with more than 2500 total subjects, the NT approach was shown to significantly reduce angina recurrence risk by 0.54 times, while revascularization risk was lowered by 0.48 times, although not statistically significant [13]. Although statistically insignificant, this might be due to the NT technique leaving the vasa vasorum intact while the CON technique might damage it, leading to a potential source of bleeding [10]. These demonstrate that both techniques are equally safe [10, 13].

According to the results of the long-term graft patency studies, a substantial difference was observed between the NT group and the CON group at 8.5 years (89% vs 76%; *p* = 0.01) and even after 16 years (83% vs 64%; *p* = 0.03) [22, 23]. The studies also highlighted that despite arterial grafts having been suggested to be superior in long-term patency compared to venous grafts, long-term patency of the NT technique was comparable to that of left internal thoracic artery grafts (83% vs 88% in 16 years) [22, 23].

**Fig. 12:**
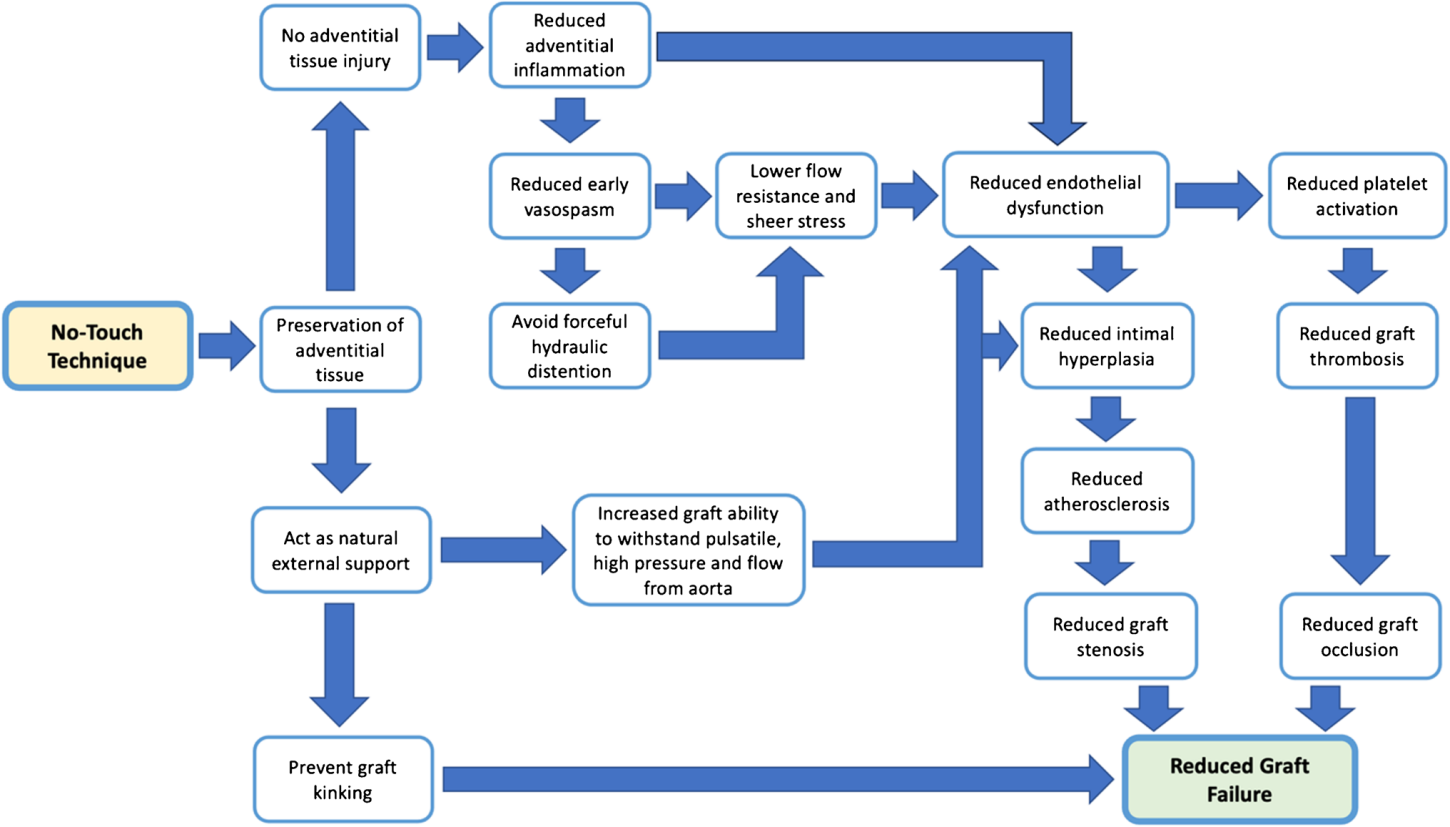
How the no-touch (NT) technique reduces vein graft failure. Preservation of adventitial tissue reduces adventitial tissue injury which reduces early vasospasm and endothelial dysfunction [8, 10, 12, 13]. No early vasospasm also means no need for forceful hydraulic distention which worsens endothelial dysfunction [8, 10, 19, 20]. Adventitial tissue also acts as a natural external support which helps the graft to adapt to hazardous blood flow from the aorta and prevents it from kinking [8, 10, 12]. All of these mechanisms that reduce endothelial dysfunction then significantly reduce graft failure [10, 13]

There are multiple possible explanations for how the NT method lowers graft occlusion and failure. First, the removal of adventitial tissue can cause adventitial damage and inflammation, leading to early vasospasm and endothelial dysfunction [10]. Intimal hyperplasia, atherosclerosis, and platelet activation are all brought on by this endothelial dysfunction, resulting in stenosis and graft occlusion [10, 13]. Second, the use of forceful hydraulic distension can be avoided as well, since the occurrence of early vasospasm is considerably reduced with the sparing of adventitial tissue in the NT approach [8, 10, 19, 20]. The same mechanism that causes endothelial dysfunction in early vasospasm can also produce endothelial dysfunction in forceful graft distension [10]. Third, the adventitial tissue serves as an organic external support system that keeps the graft from kinking and improves the vein graft’s resistance to the pulsatile, high pressure, and flow directly from the aorta, all of which can cause intimal hyperplasia, endothelial dysfunction, and other cascade events [8, 10, 12]. This is similar to how external vein stents reduce graft occlusion and failure [10, 12]. By lowering graft occlusion and stenosis, all of these processes lower the risk of angina and the requirement for revascularization [8, 10, 12, 13, 19, 20]. These mechanisms are summarized in Fig. 12.

One of the main side effects associated with the NT method is the increased incidence of leg wound complications, represented by leg infections. This is linked to the removal of the tissues around the vein and the build-up of extra cutaneous flaps, which damages more tissues and interferes with the wound’s natural healing process [8, 13]. Our analysis revealed a significant increase in leg infections in the NT group, although the results from each study were inconsistent and found no major or severe wound complications. But different results revealed the study by Pettersen et al. which demonstrated that at 6 months, the foot infection rate was lower than 5% and pretty similar in both groups [10]. This may be related to the more stringent inclusion criteria, given that the subjects in this study were all non-insulin-dependent with a normal range of body mass index [10, 12]. To prevent excess subcutaneous tissue flaps, the accuracy of the incision made just above the vein can be improved by using ultrasonography for preoperative mapping of the saphenous vein [8]. It also has been demonstrated that the NT technique has a longer learning curve compared to conventional harvesting, potentially impacting the incidence of leg complications [13].

### Limitations

Several limitations apply to our study. First, the assessed parameter remains inconsistent in some studies. The parameters other than the primary outcomes such as clinical outcomes and leg complications were not measured in all included RCTs. Leg complications were only assessed in three studies with a relatively low number of subjects, which when compared to the total sample size are significantly small (532 of total 3275 patients) [8, 10, 12, 19].

Since most trials record only the short-term follow-up, the follow-up duration is also uneven, lowering the outcome value. All follow-up durations of the RCTs included in this research are below 12 months, except studies by Souza et al. [8], Deb et al. [12], and Angelini et al. [19] which all have a relatively lower number of subjects compared to total sample size. No included studies have a follow-up length exceeding more than 18 months. Further research with a longer follow-up duration may be needed given that it has been demonstrated that graft patency may be maintained for an extended amount of time.

## Conclusion

The NT technique is proven to increase long-term graft patency with no significant risk difference for clinical outcomes compared to the CON technique. However, the leg wound complications are significantly higher in the NT technique compared to the CON technique.

## Declarations

All authors have read, reviewed, and approved the submitted manuscript to be submitted to the *Indian Journal of Thoracic and Cardiovascular Surgery*; the manuscript is original and has not been submitted elsewhere in part or whole.
